# Recommending Education Materials for Diabetic Questions Using Information Retrieval Approaches

**DOI:** 10.2196/jmir.7754

**Published:** 2017-10-16

**Authors:** Yuqun Zeng, Xusheng Liu, Yanshan Wang, Feichen Shen, Sijia Liu, Majid Rastegar-Mojarad, Liwei Wang, Hongfang Liu

**Affiliations:** ^1^ The Second Clinical College Guangzhou University of Chinese Medicine Guangzhou China; ^2^ Department of Health Sciences Research Mayo College of Medicine Mayo Clinic Rochester, MN United States; ^3^ Department of Traditional Chinese Medicine The Seventh Affiliated Hospital of Sun Yat-sen University Shenzhen China; ^4^ Department of Computer Science and Engineering University at Buffalo, The State University of New York Buffalo, NY United States

**Keywords:** education materials, patients, questions, recommendation, information retrieval

## Abstract

**Background:**

Self-management is crucial to diabetes care and providing expert-vetted content for answering patients’ questions is crucial in facilitating patient self-management.

**Objective:**

The aim is to investigate the use of information retrieval techniques in recommending patient education materials for diabetic questions of patients.

**Methods:**

We compared two retrieval algorithms, one based on Latent Dirichlet Allocation topic modeling (topic modeling-based model) and one based on semantic group (semantic group-based model), with the baseline retrieval models, vector space model (VSM), in recommending diabetic patient education materials to diabetic questions posted on the TuDiabetes forum. The evaluation was based on a gold standard dataset consisting of 50 randomly selected diabetic questions where the relevancy of diabetic education materials to the questions was manually assigned by two experts. The performance was assessed using precision of top-ranked documents.

**Results:**

We retrieved 7510 diabetic questions on the forum and 144 diabetic patient educational materials from the patient education database at Mayo Clinic. The mapping rate of words in each corpus mapped to the Unified Medical Language System (UMLS) was significantly different (*P*<.001). The topic modeling-based model outperformed the other retrieval algorithms. For example, for the top-retrieved document, the precision of the topic modeling-based, semantic group-based, and VSM models was 67.0%, 62.8%, and 54.3%, respectively.

**Conclusions:**

This study demonstrated that topic modeling can mitigate the vocabulary difference and it achieved the best performance in recommending education materials for answering patients’ questions. One direction for future work is to assess the generalizability of our findings and to extend our study to other disease areas, other patient education material resources, and online forums.

## Introduction

Diabetes is a chronic metabolic disease currently affecting almost 415 million patients worldwide with an estimation of this reaching 642 million by the year 2040 [1]. Having diabetes is associated with substantially higher lifetime medical expenditures despite being associated with reduced life expectancy [2]. Optimal control of diabetes requires a high degree of self-management where individuals have the necessary knowledge, skill, and ability for diabetes self-care [3]. Self-management consists of a complex and dynamic set of processes and is deeply embedded in each patient’s unique situation [4]. Meeting the information needs of each patient is crucial in facilitating self-management.

Patients’ self-learning is an important component of self-management. For example, through self-learning modules, patients can gain more knowledge and be more knowledgeable about practice interventions regarding foot care, which is a widely neglected part of diabetes management [5]. Meanwhile, the Internet has become an important source of self-learning for patients. Many online health communities and forums have emerged as popular platforms for patients to ask questions and share information. However, the quality of health information on the Internet is highly variable [6]. It is crucial to provide expert-vetted information to patients. At the same time, there is an abundant supply of expert-vetted patient education resources that aim to help diabetic patients improve their diabetes self-management [7-9]; however, it is quite challenging for patients without a medical background to find relevant educational materials. A system that can automatically recommend such resources to patients based on their questions in an online forum would be one way to provide relevant expert-vetted education materials.

Retrieving relevant education materials for given questions can be regarded as an information retrieval task. Information retrieval refers to the task of retrieving information of any type from a collection of documents related to search queries. One classic information retrieval approach is based on keyword matching (ie, Boolean model) [10], where documents are represented as a set of terms and queries are represented as Boolean expressions. Another popular information retrieval approach is the ranking model. Unlike the Boolean model where terms are equally weighted, the ranking model ranks the result list in terms of relevance of documents with respect to an information need expressed in the query [10]. Ranking is usually to compute numeric scores of query/document pairs where numerous scoring algorithms have been used. For example, the vector space model (VSM) computes the similarity between a query vector and a document vector, where terms can be weighted using a term frequency-inverse document frequency (TF-IDF) model [11,12]. One common idea of information seeking is to come up with good queries by thinking of words that would likely appear in a relevant document. The language models directly model such ideas where a document is a good match to a query if the document is likely to generate such a query. For a query, the probabilistic language model approach computes a probabilistic language model and ranks documents based on the probability of the model generating the query. Semantic searching intends to improve searches by understanding the semantics in queries and document collections. Concept mapping is popularly used in semantic searches where keywords are mapped to concepts captured in terminological resources. In general English, WordNet is a popular terminology resource where terms are grouped into sets of synonyms according to their meanings and organized into hierarchies based on their semantic relations [13].

Recently, topic modeling, which discovers abstract topics in document collections, has become a frequently used technique in text mining. The most common topic modeling approach is Latent Dirichlet Allocation (LDA), which allows documents to have a mixture of topics. For example, Wang and Blei [14] used topic modeling to generate an interpretable latent structure for users and items, which can provide recommendations about both existing and newly published scientific articles. In information retrieval, topic modeling can be effective in enabling the incorporation of hidden semantics [15].

In the clinical domain, there are many information retrieval applications [16], including clinical decision support. For example, InfoRetriever was designed for family medicine providers to practice evidence-based medicine [17]. Information retrieval technology is also popularly used in patient education applications, such as the PERSIVAL system, which is based on individual patient records and provides personalized access to a distributed patient care digital library by retrieving and summarizing relevant education materials.

Here, we propose a system that leverages the latest information retrieval techniques, which recommends patient education materials for questions asked by patients online. The system aims to provide expert-vetted, patient-faced information to patients. A similar system has been proposed by Kandula et al [18] where, instead of patients questions, their system recommended relevant education materials based on medical records. In this study, we investigated the use of state-of-the-art information retrieval approaches to recommend diabetes education materials for questions available in an online diabetes forum.

## Methods

**Figure 1:**
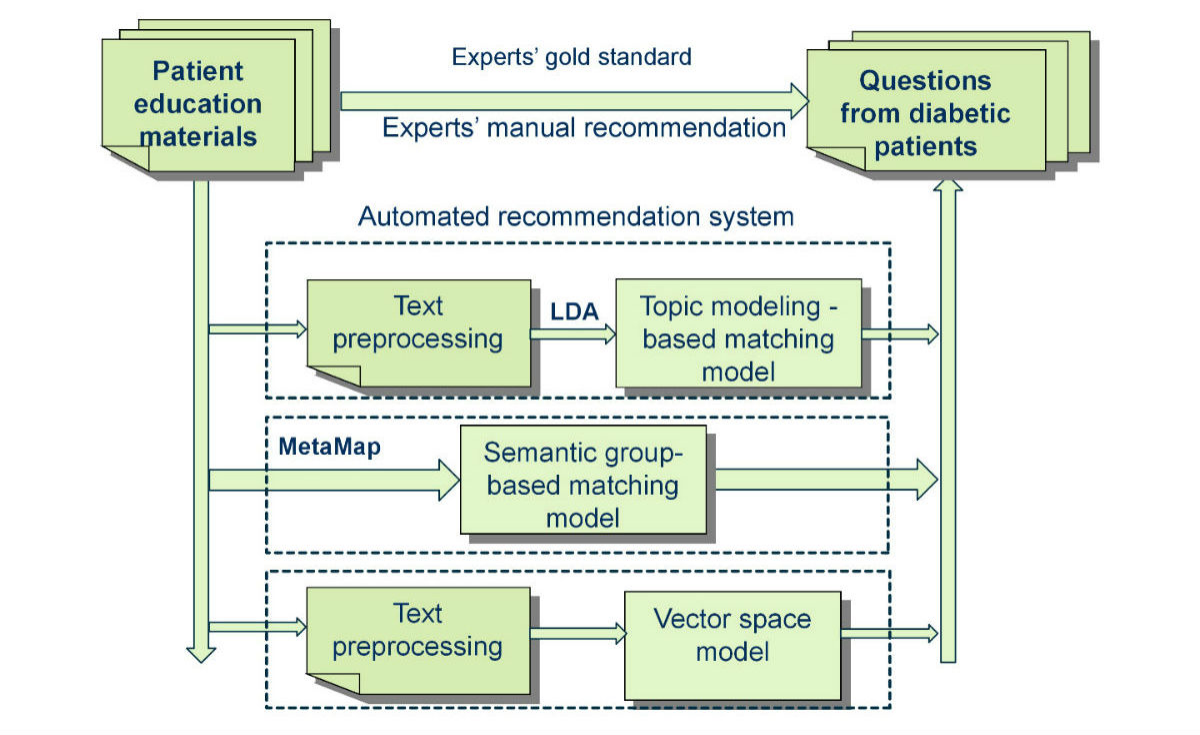
The workflow of this study.

An overview of our workflow of this study is presented in Figure 1. We designed a recommendation system using three retrieval models, including a topic modeling-based model, a semantic group-based model, and a VSM. To evaluate the performance of each model in the system, we assembled a gold standard dataset created manually for a randomly sampled subset of questions.

### Materials

The materials used for our study included a corpus of patient educational materials for diabetic patients retrieved from Mayo Clinic’s patient education database and a corpus of questions retrieved from a diabetic forum. There were more than 7400 high-quality, expert-reviewed, and outcome-based patient education materials available in the Mayo Clinic’s Database of Approved Patient Education Materials, which are indexed using disease concepts. We retrieved all diabetes-related education materials, a total of 144 documents, in PDF format and used Apache Tika, a content analysis toolkit [19], to transform the PDF format to plain text and form the patient educational materials corpus. We chose a popular diabetic online forum, the TuDiabetes forum [20], to retrieve questions asked by diabetic patients. There are more than 43,000 forum users who post questions, provide answers or comments, participate in discussions, and share experiences. Questions in the forum have been categorized into 12 categories. We gathered a total of 7510 diabetic questions from the website; for each question, the corresponding title, content, and category were extracted and formed into the corpus of questions from diabetic patients.

### Tools

We used the Unified Medical Language System (UMLS) from the US National Library of Medicine (NLM) and the associated concept-mapping tool, MetaMap, to represent and extract clinical concepts from the corpora. The UMLS is a comprehensive resource for clinical concepts, which integrates more than 2 million names for some 900,000 concepts from more than 60 families of biomedical vocabularies, as well as 12 million relations among these concepts [21]. Each clinical concept is assigned a concept unique identifier. The UMLS arranges clinical concepts into 134 semantic types. These semantic types are further grouped into 15 semantic groups. The MetaMap tool is a configurable app developed by NLM to map biomedical text to the UMLS Metathesaurus.

We used the LDA topic model with JGibbLDA software [22] to classify the patient education materials. LDA topic modeling is a common method that generates a high underlying set of topic probabilities with an infinite mixture based on a three-level hierarchical Bayesian model [23]. The statistical analysis was performed using R [24]. The attribute proportion data were analyzed using chi-square tests. We also used Cytoscape software version 3.4 to visualize the networks generated in different models [25].

### Information Retrieval Algorithms

**Figure 2:**
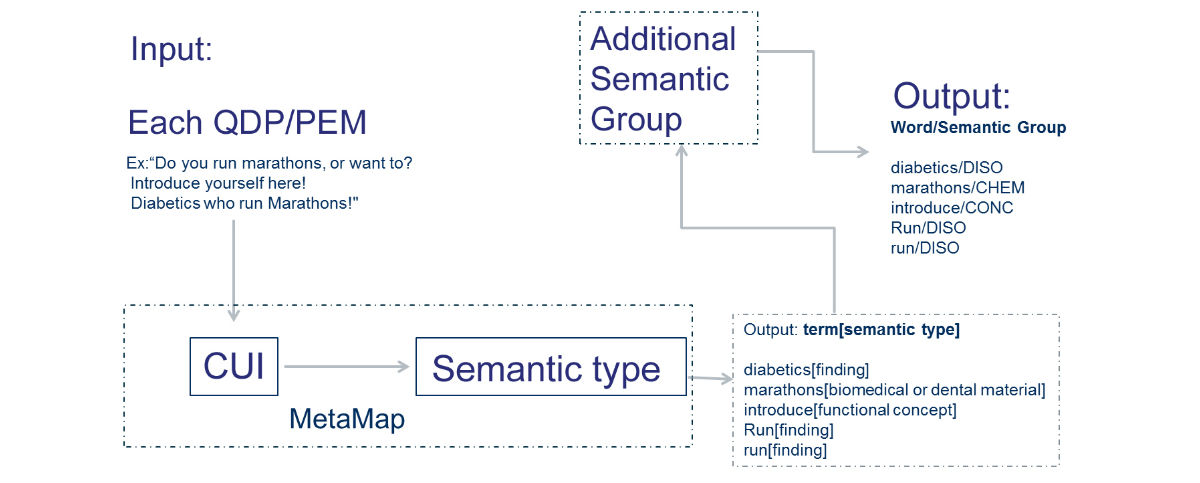
The workflow of the semantic group-based model. CHEM: chemicals and drugs; CONC: concepts and ideas; CUI: concept unique identifier; DISO: disease; QDP: questions from diabetic patients; PEM: patient educational materials.

We compared three algorithms for recommending patient education materials for matching questions: (1) a VSM model as the baseline model using scikit-learn 0.18.0 package [26], (2) a topic modeling-based matching model motivated by Kandula et al [18] using topic modeling for matching patient educational material to patient’s clinic notes, and (3) a semantic group-based matching model that considered each semantic group as a topic in the patient educational materials corpus, the detail processing in Figure 2. See Multimedia Appendix 1 for the weight calculations for the topic modeling-based and semantic group-based models.

### Gold Standard and Evaluation

To compare the performance, we randomly selected 50 questions and assembled a gold standard dataset based on manual review with the agreement of two experts. Specifically, for the pairing of question *q* and education material document *d*, we manually assigned a score in the range of 0 to 2 to indicate if d was relevant to q, where 0 indicated no relevance, 1 partial relevance, and 2 most relevance. Two medical experts performed the task. The weighted Cohen kappa value was calculated to determine interannotator agreement. A gold standard was then created based on the consensus of the two experts. The precision of the top *k* retrieved documents was used to evaluate the performance of the models, defined in the following:

Precision (k)=(number of relevant documents)/k

where a partial relevance document was counted as 0.5.

## Results

### Overall Statistics

**Figure 3:**
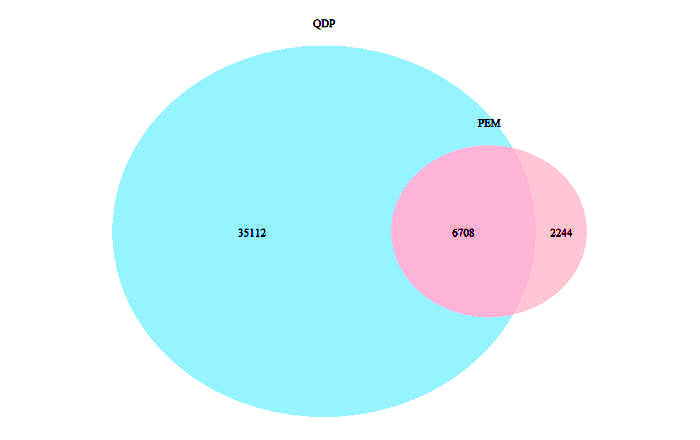
The Venn diagram of the words in the two corpora. There were 35,112 (83.96%) unique words in the questions from diabetic patients (QDP) corpus and 2244 (25.06%) unique words in the patient educational materials (PEM) corpus.

As shown in Table 1, the mean document length (word count) was 968 (SD 115) and 110 (SD 36) for patient educational materials and questions from diabetic patients, respectively. The UMLS mapping rate (the ratio of words that can be mapped to UMLS concepts) for patient educational materials was significantly higher than questions from diabetic patients (*P*<.001) with more unique concepts in questions from diabetic patients than in patient educational materials. The unique word count in questions from diabetic patients was 41,820 with 8952 in patient educational materials. The majority of the words in patient educational materials were present in questions from diabetic patients with 25.06% (2244/8952) of the words not in questions from diabetic patients (Figure 3).

**Table 1:** An overview of the two corpora.

Corpus	Number	Total word count (mapping rate)^a^	Word count, mean (SD)	Unique word count	Unique UMLS concepts, n
Questions from diabetic patients	7510	829,893 (91.18%)	110 (36)	41,820	19,616
Patient educational materials	144	139,463 (93.31%)	968 (115)	8952	7924

^a^ Mapping rate was presented the probability of words mapped to the UMLS from the total word count. Difference in mapping rate between the two corpa was statistically significant (*P*<.001).

**Figure 4:**
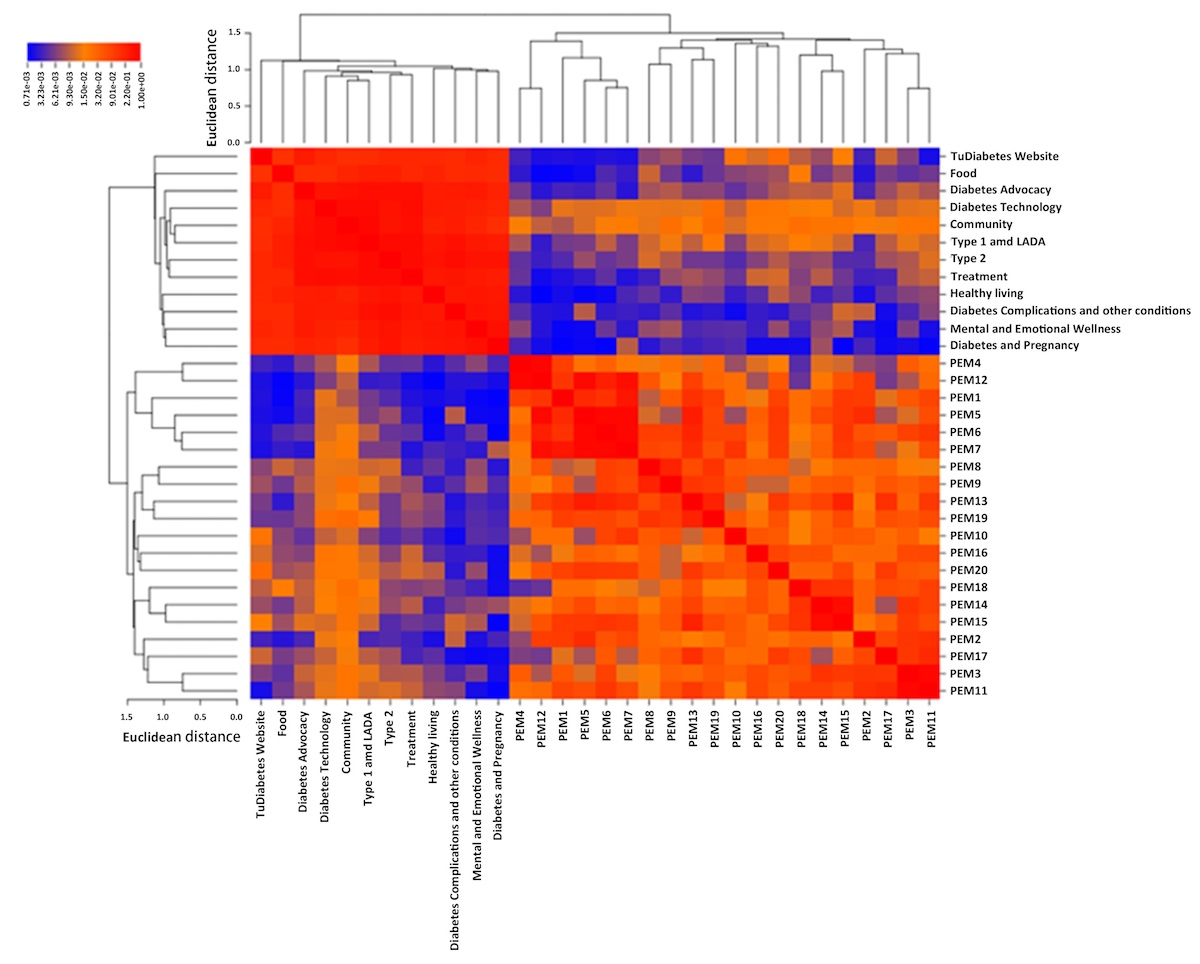
Heat map of questions from diabetic patients categories and patient educational materials topics based on cosine similarity of word vectors weighted using TF-IDF or topic word distribution. The clustering is based on Euclidean distance.

**Figure 5:**
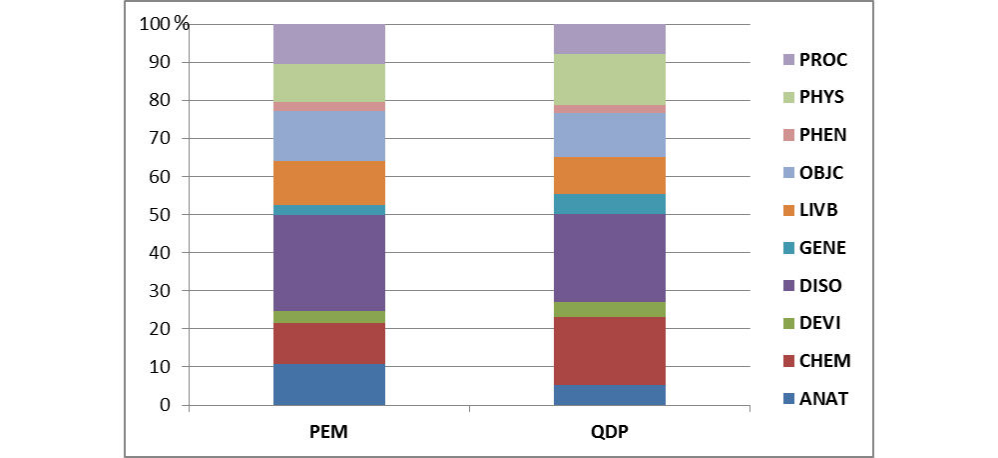
Distribution of 10 clinical semantic groups in the two corpora: questions from diabetic patients (QDP) and patient educational materials (PEM). ANAT: anatomy; CHEM: chemicals and drugs; DEVI: devices; DISO: disorders; GENE: genes and molecular sequences; LIVB: living beings; OBJC: objects; PHEN: phenomena; PHYS: physiology; PROC: procedures.

Table 2 shows the top 20 words for each corpus. The diabetes technology, community, and type 1 and latent autoimmune diabetes of adulthood (LADA) were the most common topics posted by questions from diabetic patient users, and topic 5, topic 3, and topic 8 were the main topics by topic modeling in patient educational materials documents as shown in Table 3. Table 4 shows some examples of topics obtained using topic modeling, which lists the top 20 words and their corresponding weights for each of the topics. The results of the topic vocabulary similarity analysis calculating the cosine similarity between each two topics of the two corpora are presented by a heat map graphic (Figure 4). There was no vocabulary similarity between the questions from diabetic patients categories and the patient educational materials topics, but one topic to one another topic in interior questions from diabetic patients corpus had high linguistic similarity. The semantic group distribution of the two corpora was significantly different (Figure 5) where procedures, phenomena, objects, living beings, disorders, and anatomy were more prevalent in patient educational materials, whereas physiology, genes and molecular sequences, devices, and chemicals and drugs were more prevalent in patient educational materials.

**Table 2:** The top 20 words in the two corpora.

Rank	Questions from diabetic patients		Patient educational materials	
	Word	Frequency	Word	Frequency
1	Diabetes	9062	Blood	3081
2	Insulin	5369	Insulin	2504
3	Type	4657	Glucose	2074
4	Like	4620	Diabetes	1385
5	Get	4457	Child	1166
6	Time	4107	Meal	1047
7	Know	3875	Childs	815
8	Pump	3428	Care	801
9	Now	3421	Health	797
10	Blood	3388	Dose	782
11	Day	3317	Test	738
12	People	2789	Sugar	728
13	First	2395	Help	671
14	Sugar	2383	Provider	638
15	Go	2309	Day	635
16	Back	2290	High	627
17	See	2219	Evening	583
18	Think	2148	Take	583
19	High	2088	Time	571
20	Use	2036	Eat	547

**Table 3:** Category and topic distribution of the two corpora.

Category/topic^a^		n (%)
**Questions from diabetic patients**		
	Type 2	454 (6.0)
	Type 1 and LADA	1609 (21.4)
	TuDiabetes website	97 (1.3)
	Treatment	507 (6.8)
	Mental and emotional wellness	92 (1.2)
	Healthy living	187 (2.5)
	Food	321 (4.3)
	Diabetes technology	1903 (25.4)
	Diabetes complications and other conditions	211 (2.8)
	Diabetes and pregnancy	117 (1.6)
	Diabetes advocacy	253 (3.4)
	Community	1759 (23.4)
**Patient educational materials (PEM)**		
	PEM1	6 (4.2)
	PEM2	5 (3.5)
	PEM3	13 (9.0)
	PEM4	6 (4.2)
	PEM5	15 (10.4)
	PEM6	10 (6.9)
	PEM7	3 (2.1)
	PEM8	11 (7.6)
	PEM9	5 (3.5)
	PEM10	7 (4.9)
	PEM11	9 (6.3)
	PEM12	9 (6.3)
	PEM13	6 (4.2)
	PEM14	8 (5.6)
	PEM15	3(2.1)
	PEM16	3(2.1)
	PEM17	6(4.2)
	PEM18	7(4.9)
	PEM19	5(3.5)
	PEM20	7(4.9)

^a^ The categories of the questions from diabetic patients corpus were labeled as the website provided, and the topics of the patient educational material (PEM) corpus were generated using LDA topic modeling. The topic proportion was calculated with the maximum distribution of document.

**Table 4:** Sample topics in the patient educational materials (PEM) corpus.

PEM group	Top 20 most prominent words (corresponding weight)	Topic
PEM2	Disease (0.071), kidney (0.043), risk (0.037), heart (0.031), health (0.023), pressure (0.021), care (0.018), provider (0.017), factors (0.017), people (0.017), kidneys (0.015), cholesterol (0.012), high (0.011), lifestyle (0.010), levels (0.010), protein (0.010), control (0.009), body (0.008), urine (0.008), medications (0.008)	Complication-kidney
PEM8	Food (0.039), fruit (0.024), cup (0.022), foods (0.022), eat (0.020), sugar (0.020), fat (0.019), carbohydrate (0.017), meal (0.016), plan (0.015), milk (0.015), protein (0.014), carbohydrates(0.013), snack (0.013), vegetables (0.013), grams(0.011), meals (0.011), make (0.011), calories (0.010), serving (0.010)	Food
PEM13	Care (0.024), feet (0.023), problems (0.022), provider (0.020), pain (0.020), health (0.017), term (0.017), symptoms (0.015), peripheral (0.015), website (0.014)nerves (0.013), legs (0.012), system (0.012), neuropathy (0.012), stroke (0.012), walking (0.011), figure (0.011), shoes (0.011), infections (0.009), brain (0.009)	Complication-foot

### Network Analysis

**Figure 6:**
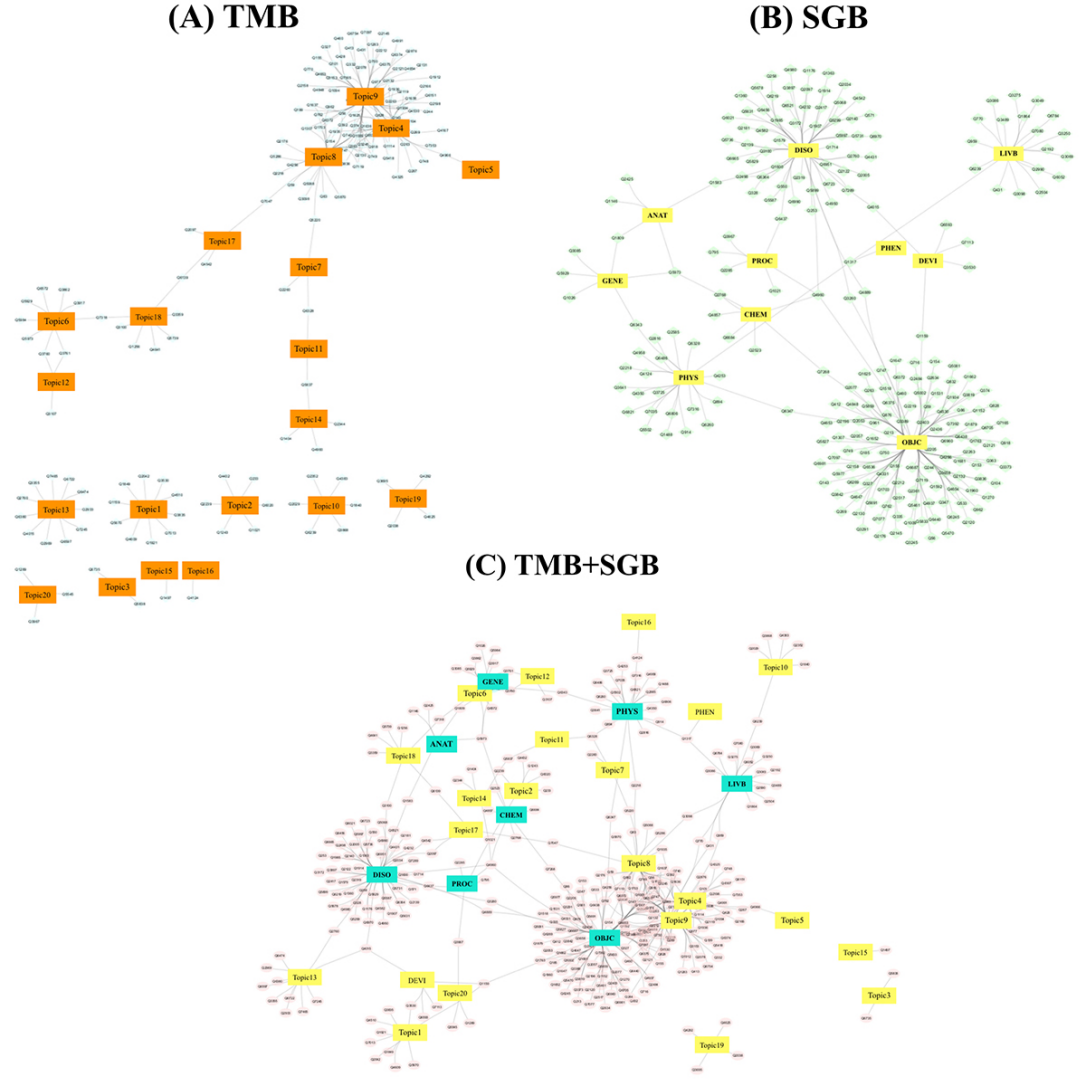
(A) Network formed using the topic modeling-based model (TMB) with topic frequency cutoff 1, (B) network formed based on the semantic group-based model (SGB) with semantic group frequency cutoff 1, and (C) a combined network by linking the two networks (TMB+SGB) based on questions.

Figure 6 shows the networks of topics or semantic groups with questions for those with the topic/semantic group frequency larger than one (ie, question 5220 matched to topic 8 with a topic frequency of 2.22, and question 4124 matched to the physiology semantic group with semantic group frequency of 4.02). In the network of topic modeling-based model (Figure 6), all patient educational materials topics were presented, there were more questions matched to topic 4, topic 8, and topic 9, whereas some topics (eg, topic 1, topic 2, topic 3, or topic 10) were relevant to a small number of questions. Some questions were associated with very specific topics. For example, question 6722 from the diabetes complication and other condition topic in questions from diabetic patients corpus, the content of the question was: “Do you have neuropathy? Introduce yourself here! Foot pain, numbness, nerve pain, does anyone else know what I’m going through? Yes, we do!” It had the unique matching to the PEM13 topic (ie, complication-foot topic). In the network of semantic group-based model (Figure 6), the objects, physiology, and live beings groups had more questions. Similarly, some questions were associated with very specific semantic groups. For example, question 7113 from the diabetes technology topic in the questions from diabetic patients corpus, the content of the question was: “Are you an Accu-Chek user? Jump in here For users of ACCU-CHEK glucose meters.” It was mapped to the devices semantic group. The combination of the two networks (Figure 6) showed that for some questions the two models, topic modeling-based and semantic group-based, were complementary to each other. For example, question 2760 belonged to the diabetes complication and other condition topic in the questions from diabetic patients corpus, the content of the question was: “Balance neuropathy I don’t have the tingling, numbness, painful neuropathy, but the feelings I have in my feet somehow aren’t being delivered to my balance center. I am having a nerve conduction test an electromyography. Any advice?” It is relevant to the complication-foot topic (ie, PEM13) and also to the disorders semantic group.

### Performance Comparison

**Figure 7:**
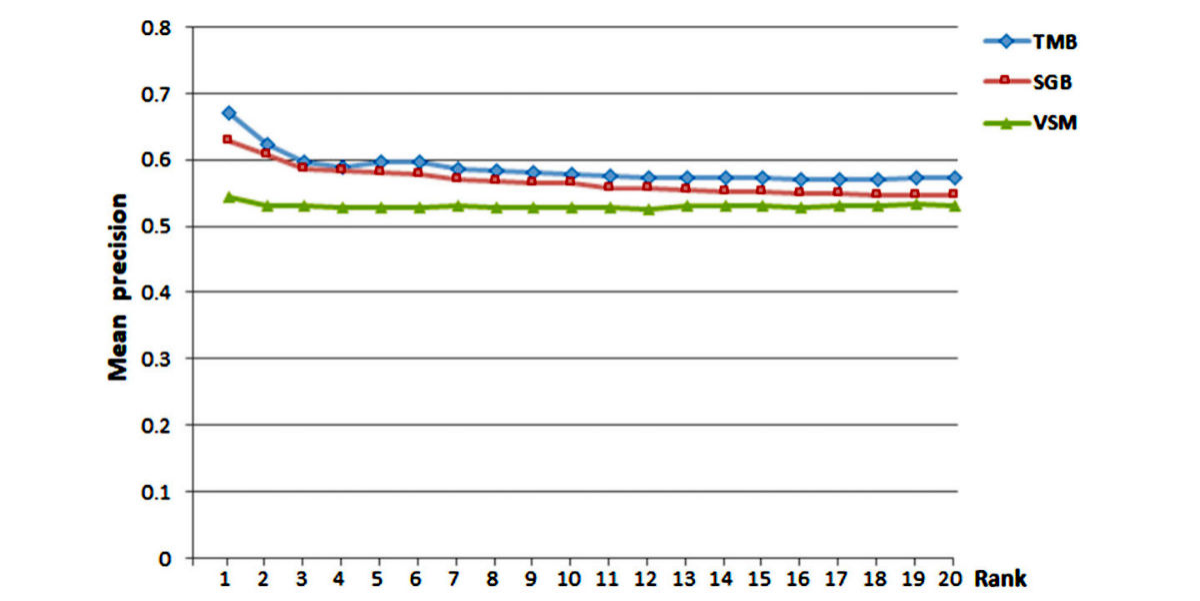
Precision at rank 1 to 20 for topic modeling-based (TMB), semantic group-based (SGB), and vector space model (VSM) models.

The two experts had a high level of agreement in relevance judgment (κ=0.90). The performance of the three models is presented in Figure 7 and Table 5. The topic modeling-based model outperformed the other two models at each rank, and the semantic group-based model had a better performance than the baseline VSM model. For example, for the top-retrieved document, the precision of the topic modeling-based, semantic group-based, and VSM models were 0.670 (67.0%), 0.628 (62.8%), and 0.543 (54.3%), respectively.

**Table 5:** Performance comparison of topic modeling-based, semantic group-based, and vector space model (VSM) models.

Model	Mean precision						
	P 1	P 2	P 3	P 4	P 5	P 10	P 20
Topic modeling-based	0.670	0.622	0.596	0.588	0.596	0.579	0.572
Semantic group-based	0.628	0.606	0.585	0.582	0.581	0.564	0.547
VSM	0.543	0.532	0.532	0.529	0.528	0.528	0.531

## Discussion

In summary, we investigated the use of the state-of-the-art information retrieval approaches to recommend diabetes education materials for questions available in an online forum for diabetes by leveraging a corpus assembled from diabetes education materials and a corpus assembled from an online forum. Our study shows that the language used in patient education materials is different from the language used in questions from an online forum. A topic modeling-based model has the potential to accurately recommend patient education material to a given question. Both topic modeling-based and semantic group-based models outperform the baseline VSM model. Network analysis illustrates that the network formed by topic modeling and the network formed by semantic groups are different and the combination of them may yield a better strategy.

Literature has shown that the language used by patients is different from the one used by clinicians [27]. Our study demonstrated that there is a language difference between patient education materials and questions in an online forum even though the target audiences of patient education materials are the patients. Patient educational materials are often produced internally by hospital staff without sufficient consideration of the patients intended to use them [28]. In our study, patient education materials tend to cover clinical and patient life topics, whereas patients tend to ask about disease-specific technology and treatment from the top words in Table 2. In addition, the semantic group of questions from diabetic patients corpus included mainly chemical drugs, physiology, devices, and gene aspects more than patient educational materials corpus, and these semantic groups also related to complication, treatment, and technology categories. There was consistency between the primary category distribution of questions from diabetic patients and their semantic groups. Therefore, analyzing online forums can identify information needs of patients and provide an opportunity to create patient-centric education materials.

The study demonstrated that topic modeling can mitigate the vocabulary difference between two corpora and achieve the best performance in recommending education materials to questions. In Figure 7, we found that the topic modeling-based model outperformed the other two models. Through topic modeling, topics and their probability distribution can be calculated for analyzing document similarity, which has been explored for document classification and personalized recommendation. For example, the iDoctor used LDA topic modeling for personalized and professionalized medical recommendation based on data available at crowd-sourced review websites [29] and Kandula et al’s [18] study also showed that the LDA topic modeling can better recommend patient education material to diabetic patients based on clinical notes. Our network analysis demonstrates that the topic modeling-based and semantic group-based models form two independent networks, which may imply that combining the two automated models has the potential to improve the recommendation.

Here, we only studied one disease and used our institutional patient education materials. More research is needed to see if our findings can be generalized. One direction for future work is to extend our study to other disease areas, other patient education material resources, and online forums.

## Appendix

Multimedia Appendix 1 Information retrieval algorithms.

## Abbreviations

LADA: latent autoimmune diabetes of adulthood
LDA: Latent Dirichlet Allocation
UMLS: Unified Medical Language System
VSM: vector space model
