# Supplementary material for: Recommending Education Materials for Diabetic Questions Using Information Retrieval Approaches
Source: J Med Internet Res. 2017 Oct 16;19(10):e342. doi: 10.2196/jmir.7754 (PMC5662791; doi:10.2196/jmir.7754)
Supplement: Multimedia Appendix 1 [file jmir_v19i10e342_app1.pdf]

Information Retrieval Algorithms

We compared three IR algorithms to recommend PEM given diabetic questions, one of which was using a VSM model as the baseline model using scikit-learn 0.18.0 package [1]. The following introduces the other two algorithms.

One is topic modeling-based matching model (TMB), inspired by *Kandula S et al* [2] where they proposed the use of topic modeling for recommending PEM to patients based on their clinic notes. After applying LDA topic modeling in the PEM corpus, we obtain the distribution of P_{w_i,t_j} for each pair of word w_i and topic t_j and the distribution of P_{t_j,d_k} for each pair of topic t_j and document d_k.

Similar to *Kandula S et al* [2], given a question q, we compute:

- $F_{w_i,q} = \frac{C_{w_i,q}}{|q|} \cdot log(\frac{|Q|}{|Q_{w_i}|})$, the TF-IDF frequency of word w_i in q. $Cw_{i,}q$ is the number of occurrences of w_i in q, $|q|$ is the number of words included in q, and $|Q|$ is the cardinality of the set of questions Q, and $|Q_{w_i}|$ is the number of questions in Q that contain w_i.
- $F_{t_j,q} = \sum_{w_i}(F_{w_i,q} \cdot \frac{P_{w_i,t_j}}{\sum_{t_j} P_{w_i,t_j}})$, the topic frequency of each topic t_j in question q. A high $F_{t_j,q}$ indicates that topic t_j is well represented in question q.
- $M_{d_k,q} = \sum_{t_j}(F_{t_j,q} \cdot \frac{P_{t_j,d_k}}{\sum_{d_k} P_{t_j,d_k}})$, the relevance of document d_k to question q. A higher value of $M_{d_k,q}$ indicates a higher relevance.

Observing most questions seeking specific types of information, such as treatment or diagnosis, we adapt topic modeling-based matching algorithm and propose semantic group-based matching model (SGB) by considering each semantic group as a topic in PEM corpus. Here, the probabilities are computed using the following equations:

- $P_{t_i,s_j} = \frac{C_{t_i,s_j}}{\sum_{t \in s_j} C_{t,s_j}}$, the probabilities of term t_i and semantic group s_j in the PEM corpus. C_{t,s_j} is the number of occurrences of t with semantic group s_j in the corpus.
- $P_{t_i,s_j} = \frac{C_{s_j,d_k}}{\sum_{s \in d_k} C_{s,d_k}}$, the probabilities of semantic group s_j and document d_k. C_{s,d_k} is the number of occurrences of s in d_k.

The relevance of document d_k to question q is computed using the same approach in TMB.

References

[1] Pedregosa F, Varoquaux G, Gramfort A, Michel V, Thirion B, Grisel O, Blondel M, Prettenhofer P, Weiss R, Dubourg V. Scikit-learn: Machine learning in Python. Journal of Machine Learning Research. 2011;12(Oct):2825-30.

[2] Kandula S, Curtis D, Hill B, Zeng-Treitler Q, editors. Use of topic modeling for recommending relevant education material to diabetic patients. AMIA annual symposium proceedings; 2011: American Medical Informatics Association. [PMID: 22195123]
